# Specific feedback makes medical students better communicators

**DOI:** 10.1186/s12909-019-1470-9

**Published:** 2019-02-08

**Authors:** Cosima Engerer, Pascal O. Berberat, Andreas Dinkel, Bärbel Rudolph, Heribert Sattel, Alexander Wuensch

**Affiliations:** 1 TUM Medical Education Center, TUM School of Medicine, Klinikum rechts der Isar, Technical University of Munich, Munich, Germany; 2 Department of General, Visceral and Transplantation Surgery, University of Heidelberg, Heidelberg, Germany; 3 Department of Psychosomatic Medicine and Psychotherapy, Klinikum rechts der Isar, Technical University of Munich, Munich, Germany; 4 CIP-Tagesklinik, Private Clinic for Psychotherapy, Munich, Germany; 5 Clinic for Psychosomatic Medicine and Psychotherapy Medical Center Freiburg, Faculty of Medicine, University Freiburg, in cooperation with Outpatient Support for Cancer Patients Comprehensive Cancer Center Freiburg, Medical Center Freiburg, Freiburg, Germany

**Keywords:** Communication skills training, CST, Specific, structured and behavior-oriented feedback, Randomized controlled trial, RCT, Medical education

## Abstract

**Background:**

Feedback is regarded a key element in teaching communication skills. However, specific aspects of feedback have not been systematically investigated in this context. Therefore, the aim of this study was to investigate the effectiveness of communication skills training (CST) integrating specific, structured and behavioral feedback.

**Methods:**

We condensed best practice recommendations for feedback in a CST for undergraduate medical students and compared the effect of specific, structured and behavior-orientated feedback (intervention group CST-behav) to general, experience-orientated feedback (CST- exp. as our control group) in a randomized controlled trial (RCT). We investigated changes on communication skills evaluated by independent raters, and evaluated by standardized patients (SP). To do that, every student was video-recorded in a pre and post assessment.

**Results:**

Sixty-six undergraduate medical students participated voluntarily in our study. Randomization did not result in equally skilled groups at baseline, so valid inter-group comparisons were not possible. Therefore, we analyzed the results of 34 students of our intervention group (CST-behav). Five out of seven domains in communication skills as evaluated by independent raters improved significantly, and there was a significant change in the global evaluation by SP.

**Conclusions:**

Although we were unable to make between-group comparisons, the results of the within group pre-post evaluation suggest that specific feedback helps improve communication skills.

## Background

Communication skills are defined as one of the core qualifications for physicians to be a medical expert [1]. Good communication reduces patient distress and confusion [2, 3], improves understanding, trust, satisfaction and compliance to treatment. [4–8] and even has positive effects on physicians themselves, such as greater job satisfaction, a reduction in workplace distress and a reduced risk of emotional burnout [3].

Systematic reviews and meta-analyses have demonstrated that communication skills can effectively be taught and learned [9–11]. They can be best acquired through practice-based training, e.g. in role-play situations with standardized patients [12–15].

Based on the results of above mentioned studies, it is of no surprise that communication skills training (CST) have meanwhile been incorporated in the educational objectives of most undergraduate medical curricula [16, 17].

Several recent studies with undergraduate medical students showed an improvement in communication skills [18], students’ self-evaluation of such skills [19, 20], and also in behavioral assessment during Objective Structured Clinical Examination (OSCE) [21]. However, there is still a lack of empirical research on how to most effectively teach communication skills so as to change students’ behavior.

In terms of general learning outcomes in medical education, the impact of feedback in a teaching context is important [22]. Van de Ridder and colleagues [22] defined feedback in clinical education as “specific information about the comparison between a trainee’s observed performance and a standard, given with the intent to improve the trainee’s performance”. One possible form of providing feedback is 360° feedback which means to receive feedback from various perspectives [23].

Despite the general notion that feedback is crucial in educational settings, including clinical education, research has shown the complexities of giving and receiving feedback and has produced equivocal results with regard to its effectiveness. For instance, while the meta-analysis by Hatala and colleagues [24] found a moderate to large effect, others report only a small effect [25]. It is pointed out that feedback can also decrease motivation and performance [25], induce shame [26] or collide with basic psychological needs and, thus, might not enhance intrinsic motivation and performance [27]. Furthermore several factors like the competence of the supervisor providing the feedback [28] can diminish the effectiveness of feedback. Van de Ridder and colleagues [29] note a lack of empirical knowledge on the variables influencing feedback communication and feedback reception.

Recently, Lefroy and colleagues [30] published consensus guidelines on the design and the content of the feedback process in clinical education, that are based on the authors’ professional experience and empirical evidence. Among others, the authors recommend to tailor the feedback to the individual trainee, reinforcing key points done well; to identify key points which might have been done better; to give specific feedback; and to ensure that the feedback is actionable. Apparently, these recommendations show clear face-validity. However, to our knowledge, such specific aspects of the feedback process have not been investigated in the context of communication skills training for undergraduate medical students.

Therefore, we conceptualized a CST for undergraduate medical students integrating current recommendations regarding feedback focusing on behavior-oriented feedback (CST-behav) and compared this to an experience-oriented feedback (CST-exp). We assessed its acceptance and its effect on self-assessed communication competence as well as objective communicative behavior. Recently, we reported that our CST was highly accepted by the participating students and improved self-assessed communicative competence in nine of 10 communication domains [31]. The aim of the current analysis was to investigate the effectiveness of this CST with regard to observable communication behavior. As a primary outcome we analyzed the observed changes in communication performance in a simulated clinical setting (video tapes) using a recently developed rating scale which was applied by independent raters [32]. As secondary outcome we assessed the change in communication skills as perceived by standardized patients. Additionally, we analyzed those subgroups of students who benefited the most.

## Methods

### Trial design

**Fig. 1:**
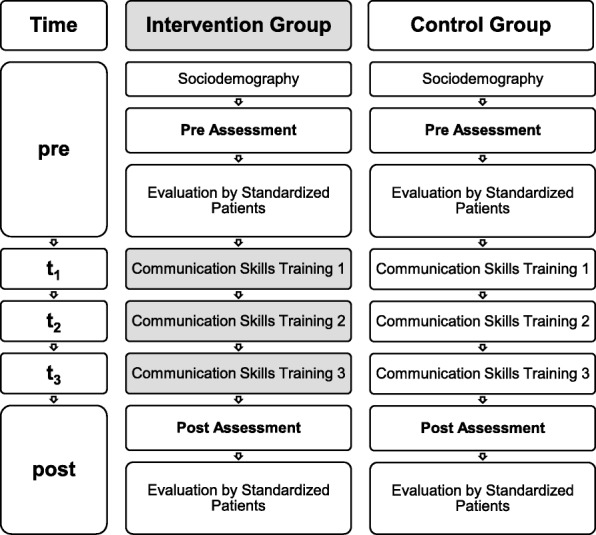
Study Design. Chronological overview of Communication Skills Training (CST) of Intervention Group with structured behavioral feedback (CST-behav) compared to CST with standard experienced based feedback (Control Group)

This study utilized a randomized trial setting to rigorously investigate the effects of a revised teaching concept for the intervention group focusing on 360° behavior-orientated feedback (CST-behav) compared to “feedback as-usual” i.e. experience-oriented feedback, for the control group (CST-exp) on observable communication skills. We applied a single center trial to one cohort of students. The study was conceptualized integrating two perspectives. Figure 1 illustrates the study design.

### Participants

The curriculum at TUM School of Medicine includes mandatory communication skills training for first clinical year students. All those students have successfully completed their preclinical studies of 2 years. All first clinical year students in winter semester 2013/14 were eligible for this study. All of them were informed about the trial, and open access to online medical books was offered as incentive for participation. Students were free to choose to participate in the clinical trial. Those who were not interested in participation received the standard CST without any formal assessment of communicative skills. Those who consented to participate signed up to one of two suitable dates for the required course. When self-selection to the course dates was finished, the course dates were randomly assigned to CST-behav and CST-exp, respectively.

### Intervention

Our mandatory CST includes three modules lasting 90 min each. The total time of the class was 270 min. The learning objective of the first module is how to begin a consultation and build up a positive relationship with the patient. The second module addresses how to structure a consultation, and the third one focuses on handling the patient’s emotions. After a brief theoretical introduction of 30 min training takes place in small groups (8–9 students) with the focus on role-play with standardized patients. Specific case vignettes are conceptualized for each module and standardized patients are prepared for these different roles. The medical students were asked to make initial contact with the patient in their role as general practitioner (GP). All students performed at least once in the role of a GP and conducted an initial contact with a standardized patient according to the case vignettes. Up to this point, training in the curriculum was experience-based, i.e. students were able to try out their communication style in order to acquire experience in communication skills. After the role-play, the fellow students, the trainer and the standardized patient provided some general feedback on the student’s communication style without specifically focusing on particular communication skills. So, feedback was based on personal and general impressions without a specific focus.

Our intervention arm (CST-behav) used the same curriculum as the control arm (CST-exp) regarding time, set-up and theoretical introduction and time for role play. However, we provided specific behavioral feedback for CST-behav, as described recently in detail [31]: First, we introduced key communication skills in a short introduction and focused on skills to initiate conversation, pick up patient’s perception, structure conversation, handle a patient’s emotions, end the conversation as well as general communication skills, e.g. taking pauses, and a global item for communicative competence. These key communication skills were summarized in a memory card and were basic for observation and feedback tasks for fellow students. Also, standardized patients (SP) and facilitating teachers were trained in giving feedback based on these skills. The feedback process was a 360° feedback and it was synchronized: All providers evaluated good and bad observed communication skills first and integrated a feed forward approach by giving suggestions on how to optimize the consultation with better communication skills. So, feedback was very specific and orientated on observable communication skills. Evaluation of the concept showed high acceptance by the students [31].

### Trainers and standardized patients

Four of the authors (POB, AD, BR, AW) were involved in the development of the new training, thus familiar with the different modes of using feedback in intervention and control group. They were assigned to deliver training to both groups (CST-exp and CST-behav): By doing so, we pursued to even out variability in each trainer’s teaching style. If we had different trainers for each group, we might have produced a bias and might have compared different teaching styles of different trainers. Due to unforeseeable circumstances, one of the control group’s courses had to be taught by a fifth, experienced trainer for one afternoon. He was instructed accordingly. Six standardized patients took part in this study and were trained appropriately for the assessment roles (video tapes) as well as for the case vignettes of the three training sessions. Due to organizational reasons the SPs were overlapping in use.

### Outcomes

The primary outcome was the performance of communication skills in a simulated physician-patient contact. The performance of communication skills was assessed by the recently validated checklist Com-ON-check [32]. This checklist uses a 5-point Likert scale ranging from 0 (poor) to 4 (excellent) for the following domains: global rating of the consultation, start of conversation, picking up patient’s perception, structure conversation, handling patients emotions, ending the conversation and general communication skills, i.e. clear wording, appropriate non-verbal communication, using suitable pausing, reinforcing questioning and checking patient’s understanding. These items were aggregated in the domain “general communication skills”. The assessment procedure for both groups (CST-behav and CST-exp) was the same for each student and was conducted before and after the intervention. Each student was asked to make initial contact as a GP with a standardized patient within a time frame of 5 min. This was videotaped and two psychologists, who were blind to group assignment and point in time, rated the consultation. Both raters first evaluated all videos independently. Where there was a discrepancy in evaluation, the raters evaluated the video once more, followed by discussion and agreement on a rating, which was later used for analysis.

Secondary outcomes were based on the evaluation by participating standardized patients. Standardized patients who were appropriately trained evaluated observed student performance with a single global item using a 10 cm long visual analogue scale [33] from poor (left side = 0) to excellent (right side = 10). Furthermore, we conducted an ancillary analysis investigating which subgroup of students benefited the most.

### Sample size calculation

Sample size was deducted from Effect Size ES estimates from our previous study [34]. There, we trained oncologists, how to communicate with patients when there is change from curative treatment to palliative treatment. In our randomized controlled trial we found moderate (*ES* = 0.61) to high (*ES* = 0.78) effect sizes for change in communication skills after CST. In order to detect an average *ES* = 0.7, with a power of 80% at a significance level of 5% (two sided t-test), 32 participants for each trial arm were required, in total. Making allowance for possible drop outs (on average, one student per group) and for a size of 9 participants for each training subgroup, we aimed to enroll 72 students. In the end, taking drop-out and taking even group assignment into account, we calculated with 66 students’ data and 34 students’ data of our intervention group CST-behav.

### Randomization and blinding

Four groups on Mondays and four groups on Wednesdays could be filled with 8 to 9 students each, resulting in 69 students approving for participation. After all students had been registered into their groups, CST-behav and CST-exp were randomly assigned by an independent person tossing a coin, with the outcome that intervention CST-behav took place on Mondays. Students and raters were unaware of the allocation process up till now.

### Statistical methods

**Fig. 2:**
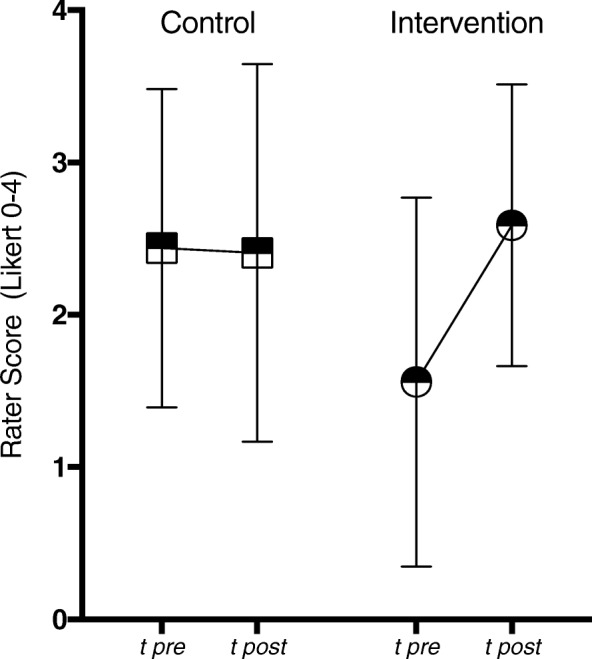
Global Rating by independent Raters of Intervention and Control Group. Data from Global Rating comparing pre with post performance (video tapes) of Intervention (CST-behav) (*N* = 34) and Control Group (CST-exp) (*N* = 32) (with 95% confidence intervals)

The participants in both arms differed – on average – significantly at baseline with regard to the primary outcome, indicated by the global rating of independent raters (see Fig. 1 and Fig. 2). As a result, we decided not to carry out the planned between-group comparisons (except for a post hoc ancillary analysis of subgroups within CST-behav and CST-exp). Consequently, our intervention group was analyzed for pre-post differences applying paired sample t-tests. Effect sizes (*ES*) were calculated using Hedges’ g [35]. An analogue analysis of the secondary outcome of the intervention group was carried out. For our ancillary analysis we made up comparable subgroups regarding to the baseline performance. We defined two groups: “low performer”: 0–1 on the global rating scale, and (moderate to) “high performer”: 2–4 on the global rating scale. The resulting sample sizes were 17 low vs. 17 high performers for the CST-behav and 6 vs. 26 for the CST-exp. A comparison of improvements in global ratings by independent raters was carried out between low and high performer within each trial group using nonparametric Mann-Whitney-U-tests.

### Ethical approval and consent

The study was approved by the Ethics Committee of the TUM School of Medicine, Munich, Germany (Project Number 5816/13).

## Results

### Participant flow and recruitment

Of 69 medical students involved in the study, three dropped out due illness. The remaining students completed the study protocol and were analyzed.

### Baseline data

The characteristics of the analyzed participants are presented in Table 1. The average age of the participants was 21.9 (*SD =* 2.0) years. The majority of the sample was female (*n* = 50, 75.8%). According to the cluster randomization, the Intervention Group (CST-behav) did not have significantly more females than the Control Group (CST-exp) (*p* = 0.20). The participants were all in a comparable phase of their studies, so almost all participants had no working experience.

**Table 1:** Sample characteristics of participants

Characteristic	Intervention Group	Control Group	p	total
N	34	32		66
age, years (M (SD))	21.4 (1.0)	22.3 (2.7)	0.28	21.9 (2.0)
sex			0.20	
male (N (%))	6 (17.6)	10 (31.2)		16 (24.2)
female (N (%))	28 (82.4)	22 (68.8)		50 (75.8)

Number, age (mean value incl. SD) and gender (N (%)) of Intervention (CST-behav), Control Group (CST-exp) and total

### Outcomes

Table 2 presents the domain-specific results of the Intervention Group (CST-behav) with the mean values of each checklist domain before and after training plus the effect sizes (*ES*). Five out of seven domains improved significantly after training, with three domains showing large effects and two medium effects. The largest improvement was in handling patient’s emotions (*ES* = 1.26).

**Table 2:** Primary Outcome of the Intervention Group (CST-behav): Observed communication performance of students evaluated by blinded raters

	Pre (SD)	Post (SD)	P^a^	ES^b^
Global rating	1.56 (1.21)	2.59 (0.92)	0.000	0.94
Start of conversation	2.06 (1.10)	1.79 (0.77)	1.93	−0.28
Patient’s perception	2.56 (0.96)	2.97 (0.72)	0.063	0.48
Structure of conversation	1.96 (1.28)	2.65 (0.92)	0.009	0.61
Patient’s emotions	1.66 (1.32)	3.10 (0.89)	0.000	1.26
End of conversation	1.52 (1.02)	2.34 (0.94)	0.003	0.82
General communication skills	2.28 (0.79)	2.75 (0.72)	0.007	0.62

Mean values incl. SD and *p*-value of each checklist domain on 5 point Likert scale, before and after training plus the effect sizes (ES)

^a^ t-test for dependent variables

^b^ Effect sizes by Hedges g

There was also a significant improvement in the secondary outcome. The global item on a 10 cm VAS evaluated by standardized patients changed significantly: *M*_*pre*_ = 6.44 *SD* = 2.28, *M*_*post*_ = 8.15 *SD* = 1.53; *p* = 0.002; This change translates into a large effect, *ES* = 1.62.

### Ancillary analysis

**Fig. 3:**
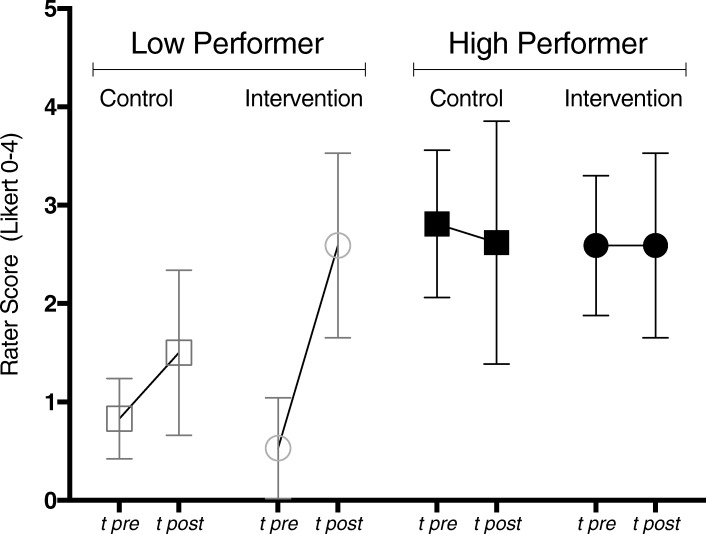
Ancillary Analysis. Global Rating of low performer vs. high performer of Intervention (CST-behav) (*N* = 34) and Control Group (CST-exp) (*N* = 32) before and after training (with 95% confidence intervals)

For an ancillary analysis we identified those students within each group who benefited the most, based on the assessments of our independent raters. In the Intervention Group (CST-behav) the low performers improved significantly from *M*_*pre*_ = 0.53 *SD* = 0.51 to *M*_*post*_ = 2.59 *SD* = 0.94; *p* < 0.001, whereas high performer remained stable: *M*_*pre*_ = 2.59 *SD* = 0.71, *M*_*post*_ = 2.59 *SD* = 0.94; *p* = 0.99. In the Control Group (CST-exp) we observed a slight improvement of low performers. However, this increase was significantly lower than that of the Intervention Group (CST-behav). Finally, high performers in the Control Group (CST-exp) even declined slightly (Fig. 3).

## Discussion

Our study investigated whether structured behavioral feedback improves the communication skills of medical students. This research question was integrated in our teaching concept, where students were asked to make initial contact as a GP with a standardized patient. The overall learning goal there focused on basic clinical communication skills, including three main topics: (1) the start of a consultation and building a trusting relationship (first module), (2) structuring a consultation (second module) and (3) addressing patient emotions (third module). A previous analysis of our data showed a high acceptance of a specific behavior-oriented feedback concept and a significant increase of students’ self-evaluation [31]. Here, we wanted to know whether behavior-oriented feedback makes a difference regarding observable communication skills.

This study demonstrates that the communication skills of medical undergraduates, trained by reference to our revised teaching concept using specific, structured behavioral feedback (CST-behav), improved significantly. Students benefited significantly pre-post in five out of six domains of defined communication competencies, and also in the global rating. We could show significant improvements for the global rating, structure of conversation, handling patients’ emotions, end of conversation and general communication skills. Start of conversation and patient perception did not change significantly. We may speculate that picking up patient perception might be a rather complex skill too difficult to assess by our standardized assessment of 5 min. Furthermore, it is very surprising that the skill start of conversation did even decrease. It might be the case that students, triggered by the intervention, paid more attention to some of the other more complex skills in the post assessment. This may be in line with the observation that students had the biggest increase in handling patients’ emotions, which is a significant challenge for medical students and even physicians [36].

The evaluation of students’ performance rated by standardized patients also changed significantly pre-post with a large effect size. The overall similar outcome from different perspectives reaffirms the significance of the intervention. Furthermore, subgroup analysis showed that the low performers in the intervention group (CST-behav) improved significantly. At the same time, the control group (CST-exp) did not produce measurable improvement pre-post.

Our results confirm with published studies on the power of feedback in different teaching contexts [29, 37]. Compared with CST by Butow and colleagues [38] and compared with the systematic review of Satterfield and Hughes [39], which both did not specifically focus on feedback in their CST, we found a high effect size on communication skills addressing emotions, whereas the review therefore points out only modest outcome effects. It seems that the didactic method of feedback, once applied in the appropriate manner, reinforces the proven substantial impact of role-plays with standardized patients on student behavioral and communicative performance [19, 40]. Our findings that low performing students benefited most of our specific behavior feedback goes in line with guidelines to feedback [30]. To our knowledge however, this has not been systematically investigated in communication skills training.

The most limiting factor in our study is that both arms revealed significantly differing initial communicative competence. This might be due to random effects of the chosen self assignments, as no other examined characteristics separate the groups. Accordingly, we decided to focus on within-group analyses for pre-post changes in each group. So, our results have to be seen critical as we cannot refer to between-group comparisons. However, as the multiperspective evaluation revealed congruent changes for the CST-behav group, a specific effect of behavior-oriented, specific feedback seems likely.

Another potential limitation was our use of a self-developed checklist [32]. Although the raters were rigorously trained to maximize internal reliability, we cannot attest to external validity of the rating scale. However, this is a common problem in communication skills research. On the one hand, Uitterhoeve and colleagues [41] pointed out the need for assessment tools closely linked to the teaching content. On the other hand, this approach to assessment limits external validity. For a deeper discussion see Radziej et al. [32].

Furthermore, the study was conducted at one single site and the participants only consisted of a selection of volunteers. These points limit the extent to which our data can be generalized. Finally, the results of ancillary analysis are based on a small sample size and results have to interpreted cautiously.

One strength of the study is the multiperspective evaluation of communication skills. Consistent findings from different perspectives strengthen the validity of the results and improves the generalizability of our results, thus ensuring objectivity: we used an objective rating of blinded raters by reference to a reliable and valid checklist for communication skills and we assessed the evaluations provided by standardized patients.

## Conclusion

Feedback can be a powerful didactic element in clinical education and communication skills training. Most educators, trainers and students will agree upon the general relevance of feedback. However, empirical evidence on the most effective way to provide feedback in order to improve communication skills in medical students is sparse. Our study is one of the first that aimed to elucidate the most effective way to provide feedback. In light of the methodological limitations, the results only tentatively suggest that specific, behavior-oriented feedback is superior to unspecific, experience-oriented feedback. We feel that our conclusion is supported by the consistent results that emerged from a multiperspective evaluation. Clearly, further research with multiple courses that are followed longitudinally are necessary in order to arrive at a firm conclusion.

## Abbreviations

CG: Control group
CST: Communication skills training
CST-behav: Communciation skills trainig with specific, structured and behavior-orientated feedback
CST-exp: Communciation skills trainig with general, experience-orientated feedback
GP: General practitioner
IG: Intervention group
RCT: Randomized controlled trial
SP: Standardized patient
